# The Impact of the Economic Recession in Greece on Assisted Reproduction Demand: A Retrospective Longitudinal Study

**DOI:** 10.3390/medicina55100654

**Published:** 2019-09-29

**Authors:** Konstantinos Sfakianoudis, Mara Simopoulou, Anna Rapani, Sokratis Grigoriadis, Evangelos Maziotis, Polina Giannelou, Agni Pantou, Terpsithea Vaxevanoglou, Maria Fakiridou, Michael Koutsilieris, Konstantinos Pantos

**Affiliations:** 1 Centre for Human Reproduction, Genesis Athens Clinic, 14-16, Papanikoli, 15232 Athens, Greece; sfakianosc@yahoo.gr (K.S.); lina.giannelou@gmail.com (P.G.); agni.pantos@gmail.com (A.P.); t.vaxevanoglou@hotmail.com (T.V.); maria_fkrd@hotmail.com (M.F.); info@pantos.gr (K.P.); 2 Department of Physiology, Medical School, National and Kapodistrian University of Athens, 75, Mikras Asias, 11527 Athens, Greece; rapanianna@gmail.com (A.R.); sokratis-grigoriadis@hotmail.com (S.G.); vagmaziotis@gmail.com (E.M.); mkoutsil@med.uoa.gr (M.K.); 3 Assisted Conception Unit, 2nd Department of Obstetrics and Gynecology, Aretaieion Hospital, Medical School, National and Kapodistrian University of Athens, 76, Vasilisis Sofias Avenue, 11528 Athens, Greece

**Keywords:** Financial crisis, Greek economic recession, In Vitro Fertilization (IVF), ART demand, Infertility treatment

## Abstract

*Background and objectives*: Since 2009, the decline of the Greek economy has been in the spotlight of the world’s financial agenda. This study assesses the economic crisis’ impact on assisted reproduction demand dynamics. *Materials and Methods*: Patient records were recruited between 2005–2017. In 2013 the clinic proceeded with a cost reduction. The studied time-frames were defined accordingly: Period A: Prior to economic crisis, Period B: During the economic crisis-prior to cost reduction, and Period C: During the economic crisis-following cost reduction. Analysis focused on parameters reflecting on patient characteristics, decision to inquire on IVF treatment, embarking on it, and proceeding with multiple cycles. *Results*: The mean annual number of first visit patients was significantly decreased during Period B (1467.00 ± 119.21) in comparison to period A (1644.40 ± 42.57) and C (1637.8 ± 77.23). Furthermore, Period C in comparison to B and A, refers to women of more advanced age (37.27 ± 5.62 vs 36.04 ± 5.76 vs 35.53 ± 5.28), reporting a longer infertility period (8.49 ± 6.25 vs 7.01 ± 5.66 vs 6.46 ± 5.20), being inclined to abandon IVF treatment sooner (2.78 ± 2.51 vs 2.60 ± 1.92 vs 4.91 ± 2.28). *Conclusions*: A decline regarding assisted reproduction techniques (ART) demand was noted as anticipated. Redefining the cost of infertility treatments may contribute towards overcoming the troubling phenomenon of postponing pregnancy due to financial concerns.

## 1. Introduction

Economic recession is a term employed to describe an extensive period of declined economic activity [1]. Since 2009 the Greek financial crisis has dominated the world’s economic agenda. The economic recession in Greece has been accompanied with unemployment, minimized wages and lower income, low inflation, and tighter credit availability. The austerity measures applied in 2010 portray probably the most drastic turning point for the country’s public sector. Gross domestic product (GDP) in 2010 was recorded at −3.5% with unemployment rates reaching 14.2% [2]. Additionally, from 2008 to 2014 GDP per capita was reduced by 32% [3]. Subsequently, the deteriorating health sector, suffered severely [4,5]. In fact the resulting poor conditions in health systems could be correlated with increased infant mortality rate and increased obstetric trauma [6].

Undoubtedly, the impact would extent to the sector of Assisted Reproduction. Literature reveals an array of studies cementing the negative correlation between economic recession and fertility [7,8]. Fertility issues stemming from poor management are irrevocably associated to economic recession. Required “cuts” were implemented as addressing infertility through treatment may not occupy the highest position on the prioritization list in comparison to other medical sectors [9]. In Greece, assisted reproduction techniques (ART) services are mainly provided by the private sector (40 licensed private in vitro fertilization (IVF) units) with a considerable contribution from the public sector (5 licensed in vitro fertilization units in general hospitals) (Greek National Authority of Assisted Reproduction).

From the patients’ perspective the psychological factor entailed regarding the economic recession should not be ignored. Uncertainty, distress, deteriorating confidence are the common denominators describing society’s perception regarding life at a time of extreme measures [7]. 

It is well established that the economic recession exerts a certain array of repercussions regarding the individual’s wellbeing and sustainably such as family planning and formation, divorces, mortality, and certainly fertility [10]. The increasing and worrisome trend of hesitant young adults postponing or overall rejecting family programming in times of recession majorly contributes to the fertility’s declined rates [11]. In addition, studies indicate that young adults institute the most sensitive and susceptible the fraction of population during recession times [12]. 

There are certain facts established regarding the Greek financial crisis hitherto. Severe unemployment and reduction of GDP have been described as the main trends [13]. Perinatal outcomes during the recession are being thoroughly investigated. An increase in low birth weight and preterm deliveries have been associated with recession, along with stillbirth rates [3]. Taking into consideration the aforementioned, a study reporting on the impact of the recession, on the decision to proceed with infertility treatment is considered timely and essential. Based on data deriving from one of the largest IVF clinic in Greece, accommodating more than 6000 IVF cycles per year (Greek National Authority of Assisted Reproduction), the authors propose to approach the matter of whether and to what extent, recession impacted the dynamics of Assisted Reproduction demand. For the purpose of this study in vitro fertilization (IVF) demand is depicted in the triptych on requiring, planning, and receiving fertility treatment. This work uniquely brings to literature data towards associating the Greek recession and infertility treatment demand-a matter examined for the first time. 

## 2. Materials and Methods 

Patients for this retrospective data analysis were recruited from medical records between 2005 and 2017. The Hospital Ethics Board approved the study protocol in accordance to the Helsinki declaration (103/6-3-2018). The study group included women diagnosed with primary infertility and subsequently submitted to IVF treatment for the first time. The inclusion criteria for recruitment were: Primary infertility diagnosis, receiving IVF treatment, permanent residence in Greece, presence of a marriage certificate or an official cohabitation agreement, first visit to the clinic between 1 Jan 2005 and 31 Dec 2017. Data collection prior to 2005 was not included as medical records were in hard copy and not in electronic format. 

The time frames for this study were divided in three major periods. Certain cut-off points defined the studied time frames. Firstly, the press conference serving to communicate to the public that Greece is entering a period of economic recession, as it was reported on the media on the 24th of April 2010. This is considered as the formal point of communicating the economic crisis to the general public. However, the acknowledgement of the economic crisis is Greece by the general public was realistically dated at the begging of the calendar year of 2010 (The Chronicle of the Great Crisis - The Bank of Greece 2008–2013). The second cut-off point is the 20% decrease applied to the cost regarding all infertility treatments in January of 2013. For the purposes of study design, the period from 1 Jan 2005 to 31 Dec 2009 is characterized as ‘’Prior to the economic crisis period’’ (Period A), the time frame from 1 Jan 2010 to 31 Dec 2012 is characterized as ‘’During the economic crisis, but prior to the cost reduction period’’ (Period B) and the time frame from 1 Jan 2013 to 31 Dec 2017 is characterized as ‘’During the economic crisis—following the cost reduction period’’ (Period C). The above mentioned time frames, were statistically compared to each other with respect to the following: The annual number of patients visiting the clinic for first time for each of all the calendar years studied, the mean annual number of patients visiting the clinic for first time throughout the two periods studied respectively, the annual number of women subjected to their first oocyte pick up (OPU) for each of all the calendar years studied, the mean annual number of patients subjected to their first OPU throughout the two periods studied respectively. Further to that, the authors reported on the mean number of years of infertility that were documented at the first visit during the two studied periods respectively. In addition, the study presents the mean age of women when they visited the clinic for first time, along with the mean age of women at their first OPU, and the mean number of completed IVF cycles documented for these patients. 

All data analyses were performed using the IBM SPSS Statistics Version 25. The comparison of the aforementioned data regarding the three studied periods was performed employing one-way ANOVA and Bonferroni correction post-hoc analysis. 

## 3. Results

**Figure 1:**
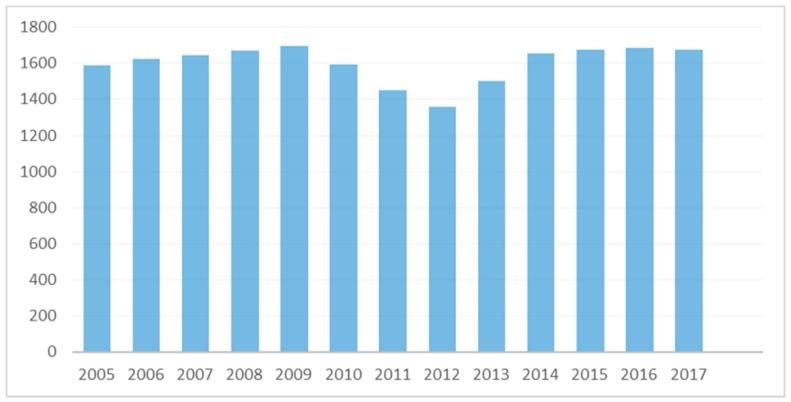
Chart on the annual number of patients visiting the clinic for the first time.

A total of 20,812 women were eligible to participate. Eight-thousand two-hundred twenty-two (8222) women who were diagnosed with primary infertility visited the Clinic for first time during Period A, 4401 women during Period B, and 8189 women during Period C, respectively. The annual number of patients visiting the clinic for the first time is presented in Figure 1. The mean annual number of patients visiting the clinic for first time during Period A was 1644.40 ± 42.57 patients/year, ranging from 1586 (in 2005) to 1696 (in 2009) patients/year. The mean annual number of patients visiting the clinic for the first time during Period B ranged from 1356 (in 2012) to 1593 (in 2010) patients/year, with an average of 1467.00 ± 119.2 patients/year. The respective mean annual number of patients visiting the clinic for the first time during Period C ranged from 1501 (in 2013) to 1685 (in 2016) patients/year, with an average of 1637.8 ± 77.23 patients/year. A statistically significant difference was established between Periods A and B (*p*-value = 0.031), as well as between Periods B and C (*p*-value = 0.038). No statistically significant difference was established between Periods A and C (*p*-value = 1).

**Figure 2:**
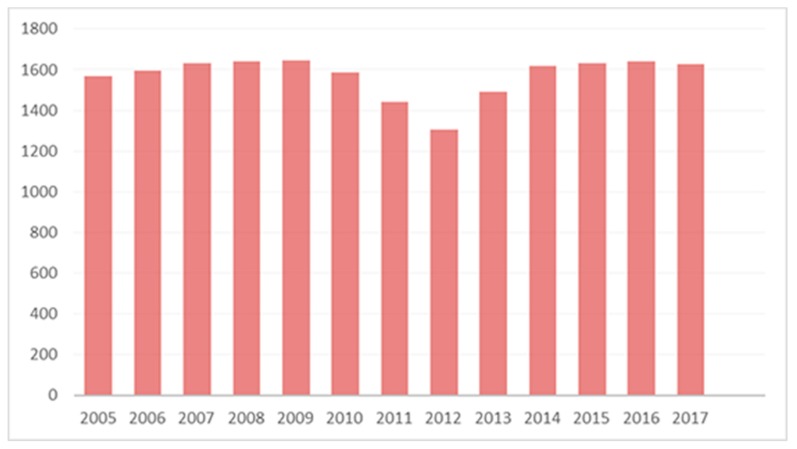
Chart on the annual number of patients subjected to a first oocyte retrieval procedure.

A total of 20,424 patients were subjected to a first OPU between 2005–2017. Eight-thousand eighty-one (8081) women were subjected to a first OPU during Period A, 4333 women during Period B, and 8010 women during Period C, respectively. The annual number of patients undergoing an OPU for the first time is presented in Figure 2. The mean annual number of patients subjected to a first OPU during Period A was 1616.20 ± 33.81 first OPUs/year, ranging from 1567 (in 2005) to 1646 (in 2009) first OPUs/year. The mean annual number of first OPUs during Period B was ranging from 1305 (in 2012) to 1585 (in 2010) first OPUs/year, with an average of 1444.33 ± 144.00 of first OPUs/year. The respective annual number of first OPUs during Period C was ranging from 1490 (in 2013) to 1640 (in 2016) first OPUs/year, with an average of 1602.00 ± 63.02 of first OPUs/year. A statistically significant difference was established between Periods A and B (*p*-value = 0.037). No statistically significant difference was established between Periods A and C (*p*-value = 1). Furthermore, evaluating the mean annual number of patients subjected to a first OPU during Periods B and C, a trend towards a difference was detected (*p*-value = 0.057).

Regarding the years of infertility reported at first visit, statistically significant differences were observed between the patients who visited the clinic for the first time during Period A and those visiting during Period B (*p*-value < 0.01) and Period C (*p*-value < 0.01). A similar trend was also observed between the patients who visited the clinic for first time during Periods B and C (*p*-value < 0.01). The statistical analysis revealed that women who visited the clinic for first time during Period C presented with a significantly longer infertility period (8.49 ± 6.25 years of infertility) in comparison to both groups of women visiting for first time during Period B (7.01 ± 5.66 years of infertility) and those visiting during Period A (6.46 ± 5.20 years of infertility). Furthermore, women who visited the clinic for first time during Period B presented with a significantly longer infertility period (7.01 ± 5.66 years of infertility) in comparison to those visiting during Period A (8.49 ± 6.25 years of infertility).

Evaluating the mean age of women visiting the clinic for the first time, statistically significant differences were also reported. Women who visited the clinic for first time during Period A were statistically significantly younger (mean age 35.53 ± 5.28) in comparison to both groups of women visiting during Period B (mean age 36.04 ± 5.76) (*p*-value < 0.01) and during Period C (mean age 37.27 ± 5.62) (*p*-value < 0.01). Furthermore, women who visited the clinic for first time during Period B (mean age 36.04 ± 5.76) were statistically significantly younger in comparison to those visiting during Period C (mean age 37.27 ± 5.62) (*p*-value < 0.01). A similar trend was also observed regarding the mean age of women at the time of a first OPU. Women subjected to a first OPU during Period A were statistically significantly younger (mean age 35.80 ± 5.64) in comparison to those subjected to OPU during Period C (mean age 37.92 ± 5.81) (*p*-value < 0.01). Moreover, women subjected to a first OPU during Period B were statistically significantly younger (mean age 36.02 ± 5.71) in comparison to those of Period C (mean age 37.92 ± 5.81) (*p*-value < 0.01). No statistically significant difference could be established following pairwise comparison between Period A and Period B regarding the mean age of women at the time of a first OPU (*p*-value = 0.903). 

With regards to the mean number of IVF cycles performed per patient during Period A, the mean number of IVF cycles was reported to be 4.91 ± 2.28 IVF cycles/patient, during Period B 2.60 ± 1.92 IVF cycles/patient, and during Period C 2.78 ± 2.51 IVF cycles/patient, respectively. A statistically significant difference could not be established following pairwise comparison between any of the abovementioned periods. 

## 4. Discussion

In order to accurately depict patients’ trends and attitudes regarding IVF treatment prior to and during the Greek economic recession, the rational of certain characterizations regarding patients should be preceded and presented. Recruiting criteria corresponding to the three time-frame groups examined, was on the grounds of acknowledging that infertility etiology referred largely to a similar spectrum of pathologies and causes throughout the years studied. Further to that, in the era of precision medicine, infertility is regarded as a highly individualized case and it should be investigated accordingly under that light. Even during economic downturns, the ability to achieve and sustain a pregnancy is primarily depended on individual characteristics. Ascertaining data on a personal level for each of our patients was infeasible due to the retrospective nature of this longitudinal study, and to the large volume of the studied population. Thus, this manuscript deciphers fertility trends on a larger mass scale. 

Our results indicate that the mean number of patients yearly referring to our clinic was significantly decreased during Period B. Following 2009, a decline in the demand of ART treatment was observed throughout the year of 2012. Interestingly, socio-economic data regarding the Greek population confirms this trend of ‘decay’ in many health sectors. In January of 2013, this realization prompted our clinic to proceed with a 20% decrease applied to cost regarding all infertility treatments. This financial intervention was remarkably depicted in our results, as the number of patients started to “recover” noting an increase. In the years following 2014, the mean number of patients annually presenting for IVF treatment was restored, reminiscing the years prior to the economic recession. 

The mean annual number of patients undergoing a first OPU is found to be decreased during Period B, in comparison to Period A. Following the cost reduction period, namely Period C, the mean annual number of patients subjected to a first OPU was increased in comparison to Period B. Further to that, women during Periods B and C presented in their first visit reporting a longer period of infertility, both prior to and following cost reduction. What may be characterized as a “hesitant” attitude on behalf of the patients during these challenging years, is also reflected by the fact that the mean age of women at the first visit appointment, and during a first OPU is found to be significantly higher than before even following cost reduction. It is evident by our results, that the age of women performing their first OPU during the economic crisis periods was advanced. Thus, these patients’ first cycle is by default compromised. This report does not focus on presenting success rates prior to and during the economic crisis period. However, our experience along with current overwhelming literature suggests that an increase in age, attributed to postponement of the first visit, may be detrimental to the treatment outcome [14]. It is a safe hypothesis that postponing infertility treatments may be largely depended on finances. Considering the anticipated pregnancy period in a couple’s life may add another level of complexity to the decision towards embarking on IVF treatment. Pregnancy comes hand in hand with an increase in expenses, while possible complications and mode of delivery in case of a cesarean section may highlight this fact [15]. 

IVF success may require the element of persistence on the patients’ behalf, as it has been voiced that at least 3 IVF cycles may be required in order to achieve a pregnancy through IVF [16]. Our results show that the mean number of attempts on IVF treatment during the economic crisis periods is reduced in comparison to the years prior to the economic crisis. However, these differences, fail to establish as statistically significant. This outcome refers to women who following a number of unsuccessful attempts of IVF treatment decided to abandon treatment. This can be interpreted based on the same persisting pattern, namely that the decline in IVF demand was an inevitable repercussion since IVF treatments are commonly accompanied with multiple cycles and unbearable costs. Hence, the period of economic crisis constitutes a challenging time for the people in need of fertility treatment. However, it is evident by the statistical analysis, that reducing the cost for performing IVF treatment could restore the demand. Nonetheless, it is noteworthy that this demand refers to women of more advanced age, presenting at their first appointment with longer infertility periods, and who are inclined to abandon IVF treatment sooner.

The nature of the study being a single-center study may pose as a limitation. However, the study is eligible to extrapolate and conclude on the initial question regarding the effect of recession on infertility treatment demand. Our clinic reports with the highest number of IVF cycles in treating infertile patients in Greece (The Greek National Authority of Assisted Reproduction). Hence, results may be viewed as representative of the Greek status in infertility treatment and demand. Further to that, the denoted decreasing trends in numbers of patients, requesting and receiving reproductive care along with the number of attempts prior to abandoning treatment may be associated to other factors acting as confounders in concluding data from this study. However, divorce, family planning challenges, marriage, and social status are equally affected detrimentally by the economic recession within similar time-frames. Nonetheless, in the presence of lack of data regarding our patients’ income, education and employment status no assumption could be attempted.

Fertility issues entail a series of psychological matters and distress that patients may experience. During the economic crisis, IVF attempts and cycles are limited due to financial challenges, thus the distress experienced by patients may be heightened, adding another level of anxiety. Modern societies face profound consequences attributed to the worldwide crisis. Therefore, the deterioration of the health care system comes as no surprise [17]. The trends revealed by interpreting the data sourced are in line with the anticipated decrease in IVF demand during the economic recession. This is evident on various levels, from the decision to inquire on IVF treatment reflected in first visit numbers, to the decision to embark on it, reflected on first OPU, extending to the decision to proceed with multiple cycles following unsuccessful attempts. What the results highlight and perhaps an element that merits further investigation and assessment from further multicenter studies, is the noteworthy positive response noted following redefinition of cost, taking into consideration the country’s current economic status. Further to that, this study clearly presents the case that a delayed and postponed first visit due to financial hurdles-experienced horizontally due to the economic recession-may detrimentally affect the chances of this special group of patients to fairly explore their fertility potential, reaching their reproductive goals. Despite the insecurity related to the possibility of embarking in a loop of costly fertility treatments in an era of economic recession, infertility matters seem to institute a priority for the majority of patients, along with overall health issues. 

## 5. Conclusions

In conclusion, with the emergence of the Greek economic recession a decline in the demand of ART treatment was observed, as prioritization of needs lead to postponement of family planning for the sub fertile population requiring infertility diagnosis investigation. Delaying or postponing a first visit to an Assisted Conception Unit, which may be attributed to the economic recession and subsequent compromised finances, may exert a negative effect with regards to the patients’ being subjected to a successful treatment. Delaying a first visit to the Assisted Conception Unit extends to a delayed first OPU on the grounds of financial strain, attributed to the economic recession. This in turn leads to patients referring for treatment at an older age, reporting with more years of experiencing infertility, and a subsequent compromised prognosis, while they tend to abandon treatment following a lower number of IVF attempts. Adjusting costs regarding infertility treatment, especially during an economic crisis, may seem to translate in patients reprioritizing family planning as evident through the restoration of ART demand.
